# Supplementary material for: Genetic comparisons of interleukin-17 reveal a framework for complex signaling evolution
Source: bioRxiv. 2026 Apr 14:2026.04.13.718218. Preprint. [Version 1] doi: 10.64898/2026.04.13.718218 (PMC13105070; doi:10.64898/2026.04.13.718218)
Supplement: Supplement 3 [file media-3.zip › Supplemental files for paper/ERC/IL17xIL17_comparisons/IL17RA_IL17RA.pdf]

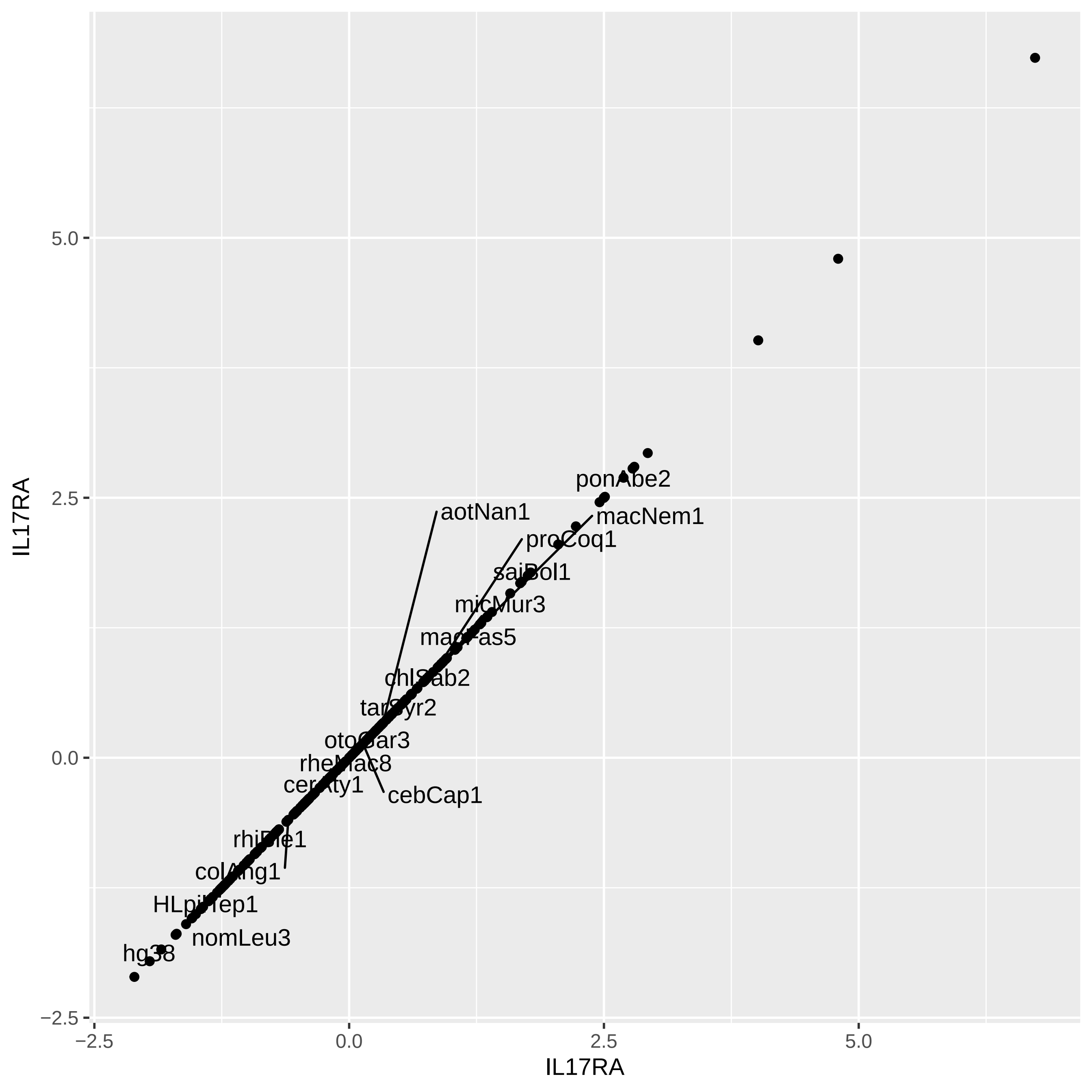
IL17RA
IL17RA
5.0
2.5
0.0
−2.5
−2.5
0.0
2.5
5.0
ponAbe2
macNem1
aotNan1
proCoq1
saiBol1
micMur3
macFas5
chlSab2
tarSyr2
otoGar3
rheMac8
cebCap1
cerAty1
rhiBie1
colAng1
HLpilTep1
nomLeu3
hg38
